# Elovl4 5-bp deletion does not accelerate cone photoreceptor degeneration in an all-cone mouse

**DOI:** 10.1371/journal.pone.0190514

**Published:** 2018-01-02

**Authors:** Christian Schori, Martin-Paul Agbaga, Richard S. Brush, Radha Ayyagari, Christian Grimm, Marijana Samardzija

**Affiliations:** 1 Lab for Retinal Cell Biology, Department of Ophthalmology, University of Zurich, Zurich, Switzerland; 2 Center for Integrative Human Physiology (ZIHP), University of Zurich, Zurich, Switzerland; 3 Department of Ophthalmology, Cell Biology and Dean McGee Eye Institute, University of Oklahoma Health Sciences Center, Oklahoma City, Oklahoma, United States of America; 4 Shiley Eye Institute, University of California San Diego, La Jolla, California, United States of America; 5 Neuroscience Center Zurich (ZNZ), University of Zurich, Zurich, Switzerland; University of Florida, UNITED STATES

## Abstract

Mutations in the elongation of very long chain fatty acid 4 (*ELOVL4*) gene cause Stargardt macular dystrophy 3 (STGD3), a rare, juvenile-onset, autosomal dominant form of macular degeneration. Although several mouse models have already been generated to investigate the link between the three identified disease-causing mutations in the *ELOVL4* gene, none of these models recapitulates the early-onset cone photoreceptor cell death observed in the macula of STGD3 patients. To address this specifically, we investigated the effect of mutant ELOVL4 in a mouse model with an all-cone retina. Hence, we bred mice carrying the heterozygously mutated *Elovl4* gene on the *R91W;Nrl*^*-/-*^ all-cone background and analyzed the retinal lipid composition, morphology, and function over the course of 1 year. We observed a reduction of total phosphatidylcholine-containing very long chain-polyunsaturated fatty acids (PC-VLC-PUFAs) by 39% in the *R91W;Nrl*^*-/-*^*;Elovl4* mice already at 6 weeks of age with a pronounced decline of the longest forms of PC-VLC-PUFAs. Total levels of shorter-chain fatty acids (< C26) remained unaffected. However, this reduction in PC-VLC-PUFA content in the all-cone retina had no impact on morphology or function and did not accelerate retinal degeneration in the *R91W;Nrl*^*-/-*^*;Elovl4* mice. Taken together, mutations in the *ELOVL4* gene lead to cone degeneration in humans, whereas mouse models expressing the mutant *Elovl4* show predominant rod degeneration. The lack of a phenotype in the all-cone retina expressing the mutant form of the protein supports the view that aberrant function of ELOVL4 is especially detrimental for rods in mice and suggests a more subtle role of VLC-PUFAs for cone maintenance and survival.

## Introduction

Stargardt-3 (STGD3, OMIM #600110) is an early onset, autosomal dominant macular degenerative disease caused by mutations in the *ELOVL4* that lead to C-terminal truncation of the ELOVL4 protein [1–4]. As a macular dystrophy, STGD3 affects mostly cones, leads to loss of central vision, decreased visual acuity and extensive formation of fundus flecks [5,6]. These clinical features are similar to other maculopathies like Stargardt-1 (STGD1) [7] and age-related macular degeneration (AMD) [8]. STGD3 discriminates from STGD1 by its autosomal dominant inheritance and from AMD by its early onset within the first few decades of life [5,6].

The ELOVL4 consists of 314 amino acids and has a 35% amino acid identity to yeast proteins of the ELO family [9]. As part of the elongase multienzyme complex, it catalyzes the initial, rate-limiting condensation reaction of the cyclical process of elongating fatty acids (FA) with 26 or more carbon atoms by adding two carbon-units to the acyl terminus per reaction cycle [10–12]. The biosynthesis of very long chain fatty acid (VLC-FA; ≥ C28) synthesis takes place in the endoplasmic reticulum (ER), where ELOVL4 is an integral membrane protein with five predicted transmembrane domains, a distinct iron binding motif, and a C-terminal di-lysine ER retention signal [1]. Three independent mutations in the last exon (exon 6) of the *ELOVL4* gene have been linked to STGD3 disease in humans [1,3,13,14]. These mutations do not affect the catalytic domain but lead to loss of function due to mislocalization of the mutant protein lacking the C-terminal ER retention signal [1,15]. In addition, mislocalized mutant ELOVL4 protein has a dominant-negative effect on the localization of wild-type (*wt*) ELOVL4 potentially by protein aggregation [15–18].

*ELOVL4* is primarily expressed in skin, testis, retina and brain where it elongates saturated (skin and brain [19–21]) and polyunsaturated (retina and testis [22–24]) FA. Very long chain saturated fatty acids (VLC-SFAs) and very long-chain poly unsaturated fatty acids (VLC-PUFAs) do not exist as free FA, but rather have their carboxyl terminus linked to a headgroup like e.g. glycerol or sphingosine to form lipids [12]. In the retina, VLC-PUFAs form glycerolipids mostly associated with docosahexaenoic acid (22:6n3; DHA) in the *sn-2* position and a polar phosphatidylcholine (PC) head group in *sn-3* [25], whereas VLC-PUFAs in the testis and VLC-SFA in the skin and brain mostly bind to sphingosine to form ceramides [19]. These ceramides are a hallmark for sperm capacitation [26] and play an important role in maintaining skin barrier functions. Thus a global *Elovl4* deletion or homozygous expression of the STGD3 causing 5 base pair (5-bp) deletion mutation leads to dehydration and neonatal lethality in mice due to lack of skin VLC-SFA that are necessary for maintenance of skin barrier [19,20,27,28]. In the adult retina, the majority of ELOVL4 is expressed in the photoreceptor (PR) cells. Only a minor portion can be found in ganglion cells [10,14,29]. Conditional PR cell specific ablation of ELOVL4 reduces the VLC-PUFA content of the retina by up to 98% without affecting retinal morphology or electrophysiological function [30]. Thus the function of the high levels of VLC-PUFAs in PR remains elusive. It has been postulated that they may play a role in the maintenance of the highly curved edges of the membrane disks of the PR outer segments (OS) by increasing membrane fluidity and support during disk shedding [11,31,32]. On the other hand, mutations in *ELOVL4*, as found in STGD3 patients, have been demonstrated to induce PR degeneration in mouse models [30,33–36].

Despite significant attempts, none of the numerous transgenic, constitutive or conditional knock-in and knock-out mouse models reproduced the early onset cone-degenerative phenotype of STGD3 patients successfully [9,20,30,33,35–38]. This may partially be based on the difficulty to analyze cone-dystrophies in the rod-dominant retina of mice.

To analyze cone cell pathophysiology without interference of the rod system, we recently generated a mutant mouse with an all-cone retina the Rpe65^R91W^;Nrl^-/-^ (*R91W;Nrl*^*-/-*^) [39]. While the ablation of the neural leucine zipper protein (NRL) transcription factor directs PR progenitors towards a cone cell fate [40], the R91W mutation in *Rpe65* reduces the content of 11-*cis*-retinal in the retina and prevents rosette formation normally observed in *Nrl*^*-/-*^ single mutant mice [39]. The resulting all-cone retina of *R91W;Nrl*^*-/-*^ is functional and has normal PR layering making this mouse suitable to study the molecular mechanisms of cone degenerations. In this study we thus investigated the effect of *Elovl4* mutations on cone PR degeneration by combining mice carrying the 5-bp deletion in *Elovl4* [33] found in STGD3 patients with *R91W;Nrl*^*-/-*^ all-cone mice [39].

## Material and methods

### Animals

Mice were housed in the animal facility of the University of Zurich and maintained in a 14 hours light: 10 hours dark cycle with access to food and water *ad libitum*. Animal maintenance and experimentation adhered to the regulations of the veterinary authorities of Kanton Zurich, Switzerland and the ARVO Statement for the Use of Animals in Ophthalmic and Vision Research. The protocol was approved by the veterinary authorities of Kanton Zurich (license nr. 109/2013 and 141/2016). The double mutant *Rpe65*^*R91W*^*;Nrl*^*-/-*^ *(R91W;Nrl*^*-/-*^) all-cone mouse line was previously generated by crossing *Rpe65*^*R91W*^ (*Rpe65*^*tm1Lrcb*^, [41]) and *Nrl*^*-/-*^ (*Nrl*^*tm1Asw*^, [40]) mutant strains, as described [39]. *129S6* (Taconic, Ejby, Denmark) and *C57BL/6J(Crl)* (Charles River Laboratories, Lyon, France) mice were used as rod-dominant *wt* controls.

The mouse harboring the 5-bp deletion (mouse_790-794_ del AACTT) in *Elovl4* (*Elovl4*^*tm1Rayy*^, [33]) was bred with the *R91W;Nrl*^*-/-*^ mouse using classical breeding schemes. The resulting *R91W;Nrl*^*-/-*^*;Elovl4*^*mut/wt*^ mice were heterozygous for the 5-bp deletion in the *Elovl4* gene and will be referred to as “*R91W;Nrl*^*-/-*^*;Elovl4*^*mut*^*”* whereas littermates homozygous for the *wt Elovl4* gene were used as controls and are referred to as “*R91W;Nrl*^*-/-*^*“*. Mice were genotyped by PCR amplification of genomic DNA from ear biopsies by specific primer pairs for *Rpe65*^*R91W*^ and *Nrl*^*-/-*^ as described earlier [39] and for *Elovl4 (fwd*: *5’-GGCCCTGTAGGAGACTGTGA-3’**; rev*: *5’-CAAAGGGTGTCACTAAACACGGCA-3’**)*.

Unless otherwise indicated, mice were euthanized by carbon dioxide asphyxiation before tissue harvesting.

### RNA isolation and semi-quantitative real-time PCR

**Fig 1:**
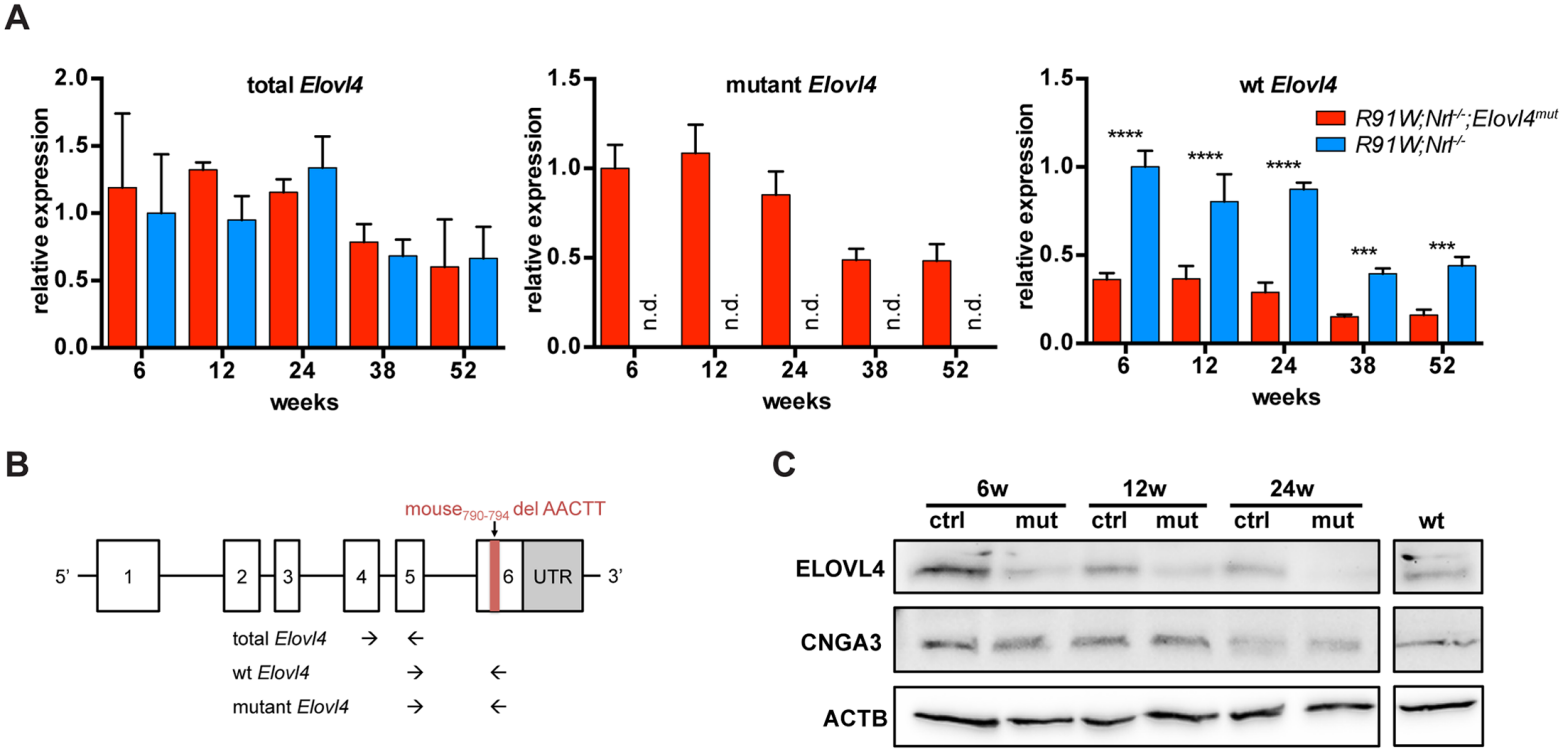
Evaluation of *Elovl4* expression in the all-cone mice. **(A)** mRNA levels of total (left), mutant (middle) and *wt* (right) *Elovl4* analyzed by semiquantitative real-time PCR. Expression was normalized to *Actb* and expressed relative to 6-week-old *R91W;Nrl*^*-/-*^ mice (left and right panel), or to 6-week-old *R91W;Nrl*^*-/-*^*;Elovl4*^*mut*^ mice (middle panel). n.d.: not detected. Shown are means ± SD. n = 3. ***: P < 0.001; ****: P < 0.0001. **(B)** Schematic representation of binding sites for primers used to discriminate between total, *wt* and mutant *Elovl4* transcripts (adapted from *Vasireddy et al*. [33]). **(C)** Western blot detection of indicated proteins in *R91W;Nrl*^*-/-*^*;Elovl4*^*mut*^ (mut), *R91W;Nrl*^*-/-*^ (ctrl) and *129S6* (*wt*, rod-dominant) mice at 6, 12 and 24 weeks of age as indicated. Shown are representative blots of n = 3.

Retinas were isolated through a slit in the cornea and snap frozen in liquid nitrogen. Total retinal RNA was isolated using an RNA isolation kit (Nucleo Spin RNA, Macherey Nagel, Oensingen, Switzerland) according to manufacturer’s instructions with an additional on-column DNAseI treatment. One microgram of RNA was used for reverse transcription by oligo(dT) and MMLV reverse transcriptase (Promega, Dübendorf, Switzerland). Semi-quantitative real-time PCR (qPCR) was performed by a LightCycler480 instrument (Roche Diagnostics, Rotkreuz, Switzerland) on 10 ng cDNA template using SYBR Green I master mix (Roche Diagnostics) and appropriate primer pairs (Table 1) designed to span large intronic regions and avoid known SNPs. For the amplification of total *Elovl4* transcripts (*wt* and mutant *Elovl4*), primers annealed to exons 4 and 5, upstream of the 5-bp deletion. For the discrimination of *wt* and mutant *Elovl4*, forward primer was designed in exon 5 and the reverse primer in the mutated or *wt* region of exon 6. Thus, only the respective form of *Elovl4* was amplified (Fig 1B). Reactions were normalized to *β-Actin* (*Actb*) and relative expression was calculated by the comparative threshold cycle method (ΔΔC_T_). n = 3 for all time points.

**Table 1:** Primers for qPCR.

**Gene**	**Fwd 5’-3’**	**Rev 5’-3’**
*Total Elovl4* ^a)^	TTTTGTATCGAAAGGCGTTG	AGGTATCGCTTCCACCAAAG
*Wt Elovl4* ^a)^	TTTGGTGGAAGCGATACCTG	ATGTCCGAGTGTAGAAGTTG
*Mutant Elovl4* ^a)^	TTTGGTGGAAGCGATACCTG	TGTATGTCCGAGTGTAGGAG
*Gnat2*	GCATCAGTGCTGAGGACAAA	CTAGGCACTCTTCGGGTGAG
*Arr3*	CTGGCACTGGATGGCAAACT	CCCCATAGGACACCACCAAG
*Actb*	CAACGGCTCCGGCATGTGC	CTCTTGCTCTGGGCCTCG

^a)^ described in *Vasireddy et al*. [33]

### Immunofluorescence

Mice were anesthetized with a lethal dose of ketamine/xylazine cocktail and transcardialy perfused with 4% (w/v) paraformaldehyde (PFA) prepared in phosphate buffer (0.1 M, pH 7.4). Eyes were marked nasally, enucleated and processed for cryosectioning, as described earlier [42]. Dorsoventral sections (12 μm) were blocked for 1 hour with 3% normal goat serum (containing 0.3% Triton X-100 in PBS), and incubated overnight at 4°C with rabbit anti-ELOVL4 antibody [11] (1:200), or *A*. *hypogaea* PNA-FITC (1:250, L7381, Sigma, Buchs, Switzerland). After washing, slides were incubated with secondary anti-rabbit antibodies labeled with Cy3 (Jackson ImmunoResearch Laboratories, Soham, UK), counterstained with 40,6-diamidino-2-phenylindole (DAPI), and analyzed by fluorescence microscopy (Zeiss, Axioplan, Jena, Germany). n = 3 for all time points.

### Western blotting

Retinas were isolated through a slit in the cornea and homogenized by sonication in 200 μL of 100 mM Tris/HCl (pH 8,0). Homogenates were centrifuged and protein contents in the supernatants determined using Bradford reagent (Bio-Rad, Hercules, CA, USA). SDS–PAGE and Western blot analysis were performed as described earlier [42] using the following primary antibodies: rabbit anti-C-terminus of ELOVL4 [11] (1:1’000), rabbit anti-cone photoreceptor cGMP-gated channel subunit alpha (CNGA3) [43] (1:500) and mouse anti-ACTB (1:10’000, A5441, Sigma). Immunoreactive signals were detected using the Western lightning chemiluminescence reagent (PerkinElmer, Waltham, MA, USA) and visualized using the Fusion FX chemiluminescence & fluorescence imaging system (Vilber Lourmat; Collégien; France). n = 3 for all time points.

### Retinal FA and phospholipid composition

Retinas were isolated through a slit in the cornea, snap frozen in liquid nitrogen and stored at -80°C until analysis. For FA profiles, total lipids were extracted following the method of Bligh and Dyer [44] with modifications [45]. To each lipid extract 15:0 and 17:0 FAs were added as internal standards. The lipid extracts were subjected to acid hydrolysis/methanolysis to generate fatty acid methyl esters (FAMEs) [46]. FAMEs were quantified using a gas chromatograph (6890N, Agilent Technologies, Foster City, CA, United States) with flame ionization detector (GC-FID) [47]. Data is represented as relative mole percent of each FA species. Additionally, the retinal phospholipid composition was analyzed by methods described previously [21,48]. Briefly, retinas were homogenized in methanol and diluted 1:40 with 2-propanol/methanol/chloroform (4:2:1 v/v/v) containing 20 mM ammonium formate and 1.00 μM PC 14:0/14:0, 1.00 μM phosphatidylethanolamine (PE) 14:0/14:0 and 0.33 μM phosphatidylserine (PS) 14:0/14:0 as internal standards. Samples were introduced into a triple quadrupole mass spectrometer (TSQ Ultra, Thermo Scientific, Waltham, MA, United States) using a chip-based nano-ESI source (NanoMate, Advion, Ithaca, NY, United States) operating in infusion mode. PC lipids were measured using precursor ion scanning of *m/z* 184, PE lipids were measured using neutral loss scanning of *m/z* 141, and PS lipids were measured using neutral loss scanning of *m/z* 185. Quantification of lipid molecular species was performed using the Lipid Mass Spectrum Analysis (LIMSA, University of Helsinki) software’s peak model fit algorithm. Data is represented as the relative percent of each measured species within each class (PC, PE, PS) ± standard deviation. n = 4 for all time points.

### Morphology and nuclei count

Eyes were marked nasally, enucleated, fixed in glutaraldehyde (2.5% in cacodylate buffer) overnight at 4°C, trimmed, post-fixed in 1% osmium tetroxide and embedded in Epon 812 as described [42]. Dorsoventral semithin cross-sections of 0.5 μm were cut through the optic nerve head, stained with toluidine blue and analyzed using light microscopy (Zeiss). Images of higher magnifications were acquired from the central region close to the optic nerve head. PR nuclei were counted manually in the region at 200–400 μm from the optic nerve head. n = 3 for all time points.

### Electroretinography

Electroretinograms (ERG) of 6- (n = 5), 12- (n = 6), 24- (n = 6), 38- (n = 4) and 52- (n = 3) week old mice were recorded using an LKC UTAS Bigshot recording unit (LKC Technologies, Inc. Gaithersburg, MD, USA) as previously described [49]. In brief: Mice were dark adapted overnight and pupils dilated with Cyclogyl 1% (Alcon Pharmaceuticals, Fribourg, Switzerland) and Neosynephrine 5% (Ursapharm Schweiz GmbH, Roggwil, Switzerland) 20 min before recording. Mice were anesthetized by subcutaneous injection of Ketamine (85 mg/kg; Parke-Davis, Berlin, Germany) and Xylazine (4 mg/kg Bayer AG, Leverkusen, Germany). The cornea was kept moist with a drop of Mydriaticum dispersa (OmniVision AG, Neuhausen, Switzerland). Recording gold electrodes were placed directly onto the cornea of each eye, a reference electrode was inserted under the skin between the eyes and a grounding electrode was positioned subcutaneously at the tail base. All manipulations were performed under dim red light. ERG responses were recorded after adapting the eyes to low background light (30 cd × s × m^-2^) for 5 min. Responses to single white light flashes with intensities ranging from -10 to 25 dB (0.25 to 790 cd × s × m^-2^) divided in 8 steps were recorded with interstimulus intervals of either 5 seconds (for –10, –5, –0, and 5 dB), or 17 seconds (for 10, 15, 20, and 25 dB). Ten responses per light intensity were averaged.

### Statistical analysis

Statistical analysis was performed using Prism 6 software (GraphPad, San Diego, CA, USA). All data are presented as mean values ± standard deviation (SD). The number of samples (n) used for individual experiments is given in the methods section. Two-way ANOVA with Holm-Šídák correction for multiple comparisons was used to determine statistical significance. P-values below 0.05 were considered significant (< 0.05: *; < 0.01: **; < 0.001: ***; < 0.0001: ****).

## Results

### Expression of wt and mutant Elovl4 in the R91W;Nrl^-/-^ retina

Cones are the most affected PR type in the retina of STGD3 patients. We thus investigated the effects of a disease-linked *Elovl4* mutation on cones using our *R91W;Nrl*^*-/-*^ all-cone mouse model. The *R91W;Nrl*^*-/-*^*;Elovl4*^*mut*^ mouse was generated to express a *wt* and mutant copy of *Elovl4*, mimicking the autosomal dominant inheritance of STGD3 macular degeneration in all cone PR cells of *R91W;Nrl*^*-/-*^ background. To detect potential differences in the expression of *wt* or mutant *Elovl4* mRNA, we compared retinal levels of *Elovl4* transcripts between *R91W;Nrl*^*-/-*^*;Elovl4*^*mut*^ and *R91W;Nrl*^*-/-*^ mice (Fig 1A and 1B). The total transcription levels (primer pair recognizing both *wt* and mutant *Elovl4*) were similar between control and mutant mice, indicating a continuous transcription of both forms of *Elovl4*. The general reduction of *Elovl4* transcript levels in aged *R91W;Nrl*^*-/-*^*;Elovl4*^*mut*^ and *R91W;Nrl*^*-/-*^ mice was most likely due to the slow intrinsic retinal degeneration in the *R91W;Nrl*^*-/-*^ mouse itself [39]. This goes along with the observed age-dependent decline of cone-specific transcript levels such as G protein subunit alpha transducin 2 (*Gnat2*) and retinal cone arrestin-3 (*Arr3*), whereas expression of marker genes for bipolar and ganglion cells did not decrease during the whole time course (S1 Fig). As expected, the mutant *Elovl4* transcript was detected exclusively in *R91W;Nrl*^*-/-*^*;Elovl4*^*mut*^ mice (Fig 1A, middle panel), whereas levels of the *wt Elovl4* transcript were reduced at all time points in mutant mice (Fig 1A, right panel).

Analysis of the ELOVL4 protein by Western blotting, using an antibody specific for C-terminus, confirmed reduced levels of the *wt* ELOVL4 protein in *R91W;Nrl*^*-/-*^*;Elovl4*^*mut*^ mice compared to their respective controls throughout all time points. In contrast, signal intensities for the cone-specific protein CNGA3 were similar in *R91W;Nrl*^*-/-*^ (control) and *R91W;Nrl*^*-/-*^*;Elovl4*^*mut*^ mutant mice at individual time points (Fig 1C). However, as for ELOVL4, a reduction in CNGA3 signal was detected in both mouse models during ageing (Fig 1C).

### Localization of ELOVL4 protein in the all-cone retina

**Fig 2:**
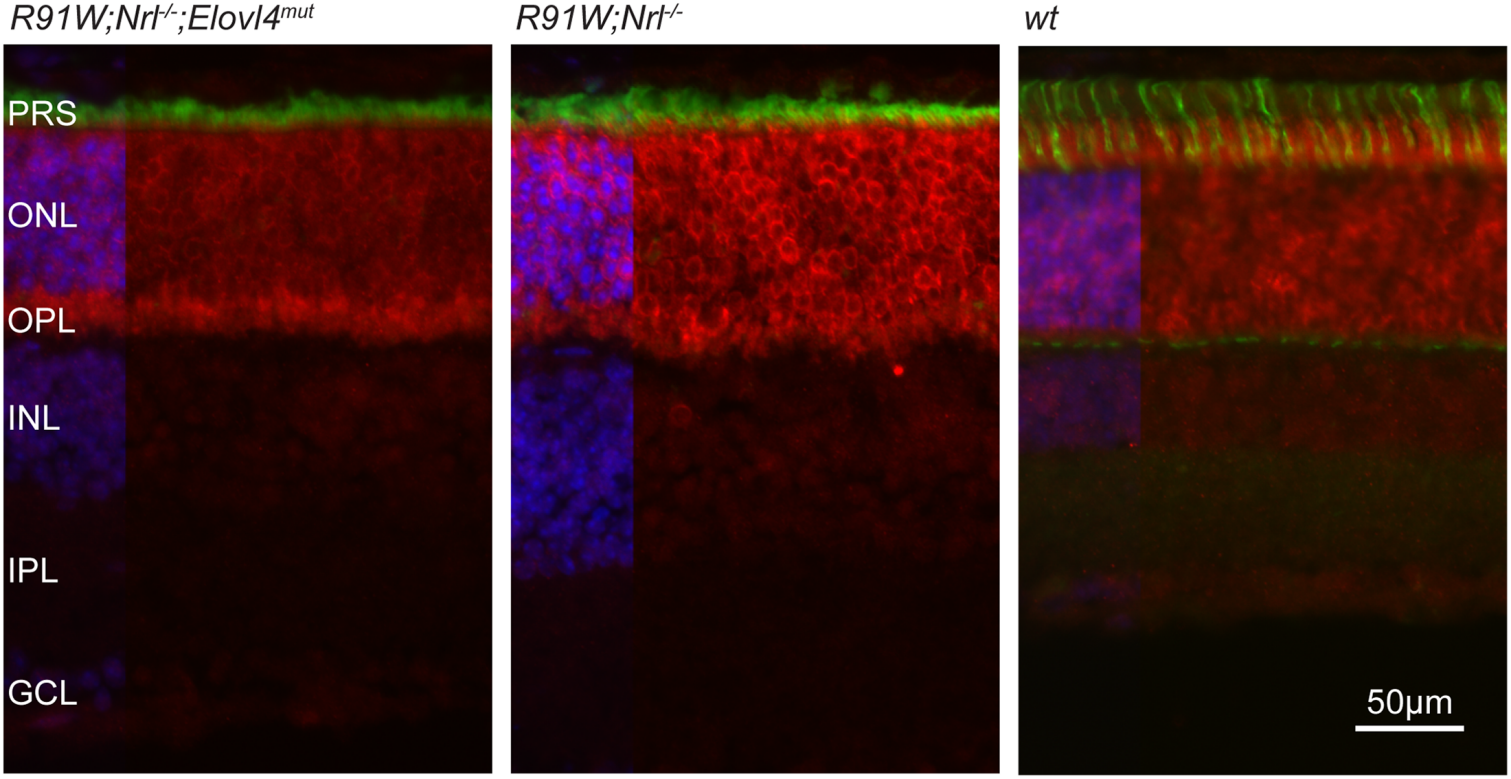
ELOVL4 distribution in *R91W;Nrl*^*-/-*^*;Elovl4*^*mut*^, *R91W;Nrl*^*-/-*^ and C57BL/6J (*wt*) rod-dominant retina of 12-week-old mice. Red: ELOVL4; green: PNA (cone PR segments); blue: DAPI. PRS: photoreceptor segments; ONL: outer nuclear layer; OPL: outer plexiform layer; INL: inner nuclear layer; IPL: inner plexiform layer; GCL: ganglion cell layer. Scale bar: 50 μm. Shown are representative images of n = 3.

ELOVL4 protein is highly expressed in the mouse retina and predominantly localized to the ER of both rod and cone PR cells [14]. We detected strong staining in the outer nuclear layer (ONL) and very weak signals in the inner nuclear layer (INL) and ganglion cell layer (GCL) (Fig 2). Co-staining of ELOVL4 with PNA indicated the absence of ELOVL4 signal from the OS of cones in mice of all genotypes (Fig 2). No major differences in tissue distribution of *wt* ELOVL4 immunoreactivity was observed between *R91W;Nrl*^*-/-*^*;Elovl4*^*mut*^, control *R91W;Nrl*^*-/-*^ as well as C57BL/6J (*wt*) wild type mice. The overall staining intensity in the ONL, and occasionally observed intense staining at the endfeeds of PR close to the outer plexiform layer (OPL), was variable and could not be assigned to a specific genotype (Fig 2) or age group (not shown).

### Analysis of retinal FA composition

ELOVL4 catalyzes the rate limiting condensation reaction which is responsible for the elongation of saturated and poly-unsaturated C26 to C28 FA and is postulated as well to be the responsible desaturase for all further elongation steps from C28 –C40 and beyond [11,18]. In the retina, VLC-PUFAs form phospholipids by binding to the *sn-1* position of PC, whereas DHA is bound to *sn-2* [12].

**Fig 3:**
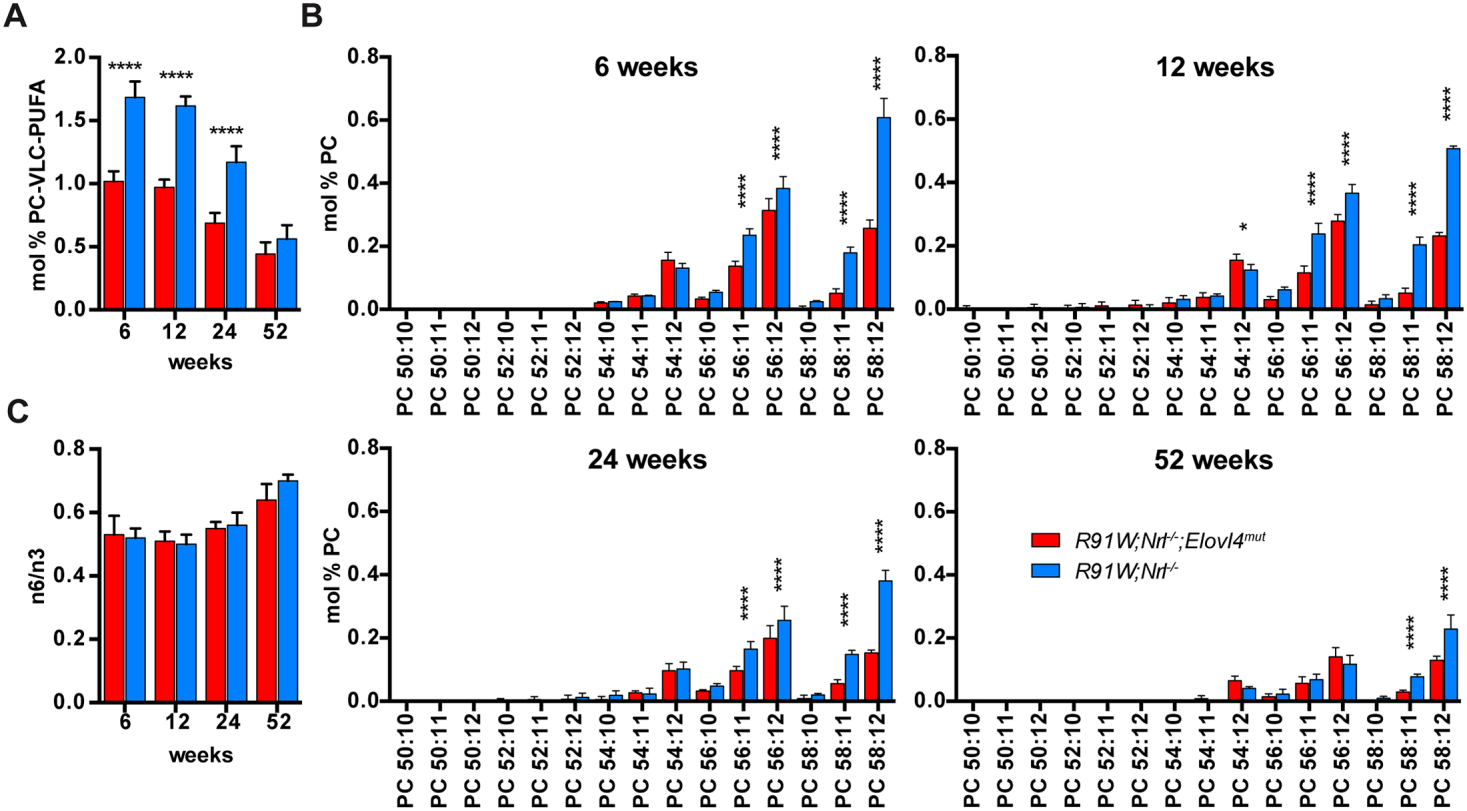
Retinal FA composition. **(A)** Percentage of the sum of PC-VLC-PUFAs in the total PC pool at indicated ages. **(B)** All individually identified VLC-PUFAs of the PC pool at indicated ages. The glycerolipids are composed of a PC headgroup, a DHA (22:6) and a VLC-PUFA (e.g.: PC-58:12 (22:6/36:6)) **(C)** Ratio of n6 to n3 FA in total lipids at indicated ages. Shown are means ± SD (n = 4; *: P < 0.05; ****: P < 0.0001).

Expression of the mutant *Elovl4* strongly affected the PC-VLC-PUFA composition already in young animals. At 6 weeks of age, the total VLC-PUFAs of the PC fraction (28:4 to 36:6) were reduced by 39% in *R91W;Nrl*^*-/-*^*;Elovl4*^*mut*^ compared to corresponding all-cone control mice (Fig 3A). This is in agreement with the reduced ELOVL4 protein and transcript levels observed by Western blotting and qPCR, respectively. The reduction of PC-VLC-PUFAs in *R91W;Nrl*^*-/-*^*;Elovl4*^*mut*^ was most pronounced for the longest PUFAs (Fig 3B). For example, while in 12-week-old *R91W;Nrl*^*-/-*^*;Elovl4*^*mut*^ mice, the PC-54:11 (PC-22:6/32:5) showed only a tendency to reduced levels when compared to *R91W;Nrl*^*-/-*^ *control* mice, the PC-VLC-PUFAs were reduced by 52% for the 56:11 (22:6/34:5) and by 75% for 58:11 (22:6/36:5). As expected, since ELOVL4 is not involved in biosynthesis of C18—C24 fatty acids, mutation of one allele of *Elovl4* did not affect the composition of C18—C24 polyunsaturated FA, hence the levels 20:4n6, and 22:6n3 were not affected 6 (S1 Table). Also the omega-6:omega-3 (n3/n6) ratio of PUFAs was comparable in the two mouse strains and did not change over time (Fig 3C).

### Morphology and electroretinography

**Fig 4:**
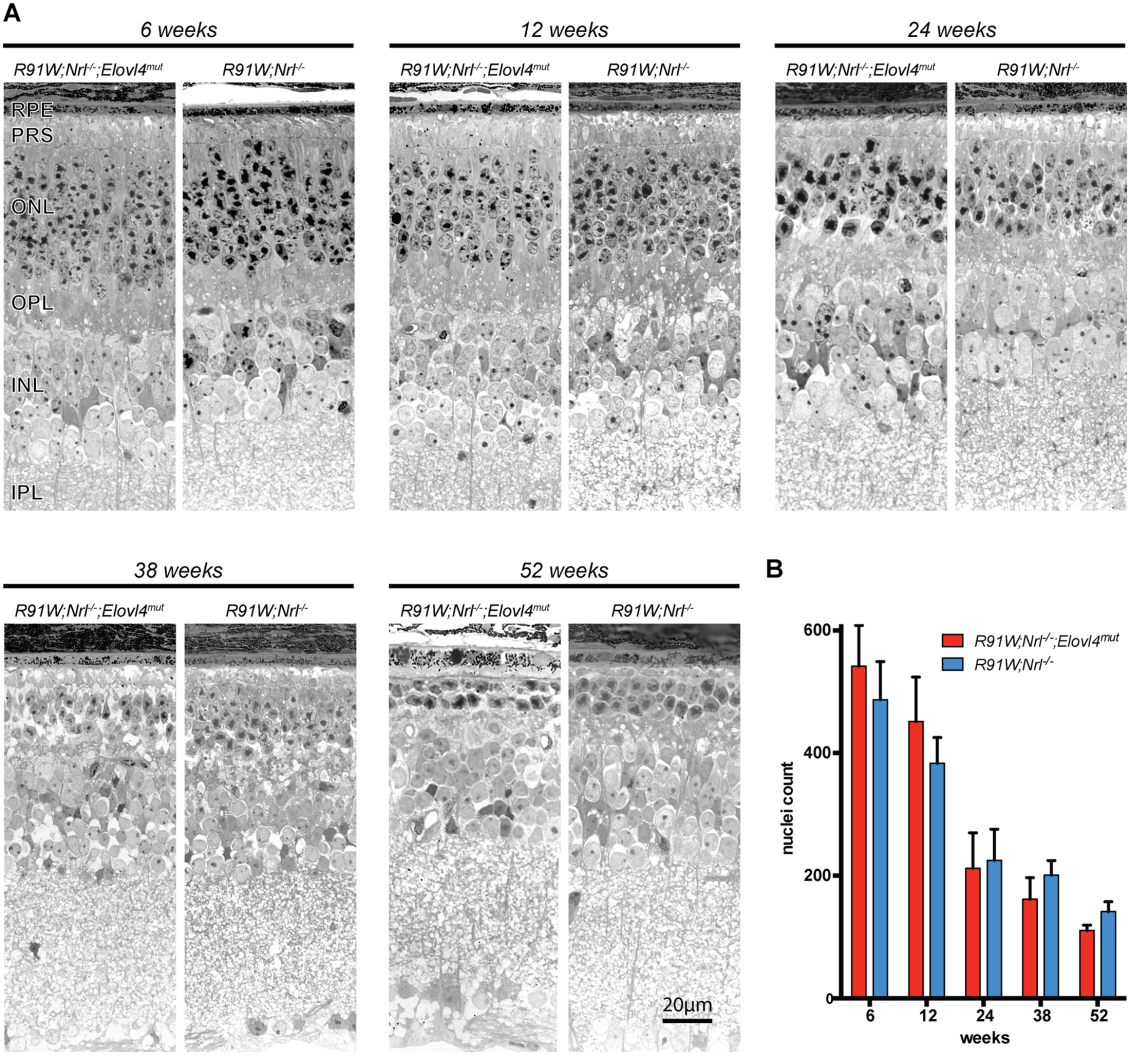
Analysis of retinal morphology. **(A)** Representative micrographs of retinas from *R91W;Nrl*^*-/-*^*;Elovl4*^*mut*^ and *R91W;Nrl*^*-/-*^ mice at indicated ages. **(B)** Cone nuclei count in the central retina. RPE: Retinal pigment epithelium, other abbreviations as in Fig 2. Red bars: *R91W;Nrl*^*-/-*^*;Elovl4*^*mut*^ mice; blue bars: *R91W;Nrl*^*-/-*^. Scale bar: 20 μm. Shown are means ± SD (n = 3).

Based on the data presented for the original mouse model of STGD3 (*Elovl4*^*tm1Rayy*^, [33]), a degenerative phenotype in *R91W;Nrl*^*-/-*^*;Elovl4*^*mut*^ mice was expected. However, retinal morphologies did not indicate accelerated degeneration in *R91W;Nrl*^*-/-*^*;Elovl4*^*mut*^ mice (Fig 4). None of the analyzed points (latest time point was 1 year of age), showed a qualitative (Fig 4A) or quantitative (Fig 4B) difference in retinal thickness between the two strains.

**Fig 5:**
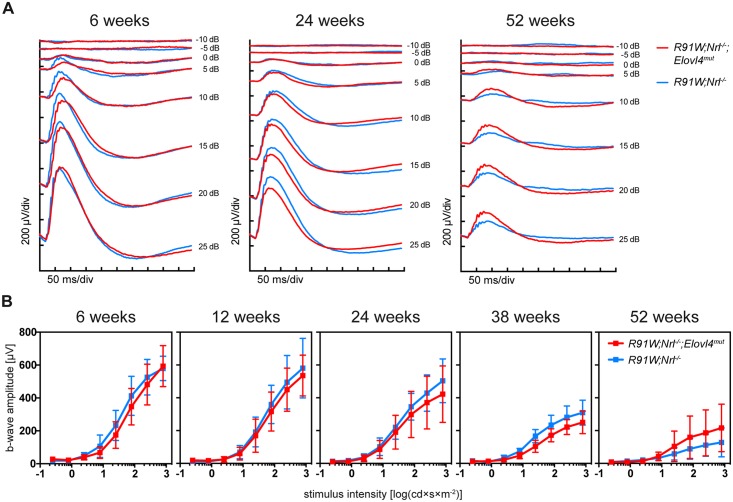
Light-adapted ERG to test retinal function. **(A)** ERG traces of early (6 w), intermediate (24 w) and late (52 w) time points. **(B)** Photopic b-wave amplitudes plotted against the logarithm of light intensities in *R91W;Nrl*^*-/-*^*;Elovl4*^*mut*^ and control *R91W;Nrl*^*-/-*^ mice at indicated ages. Shown are means ± SD (6 weeks, n = 5; 12 weeks, n = 6; 24 weeks, n = 6; 38 weeks, n = 4; and 52 weeks, n = 3).

Additionally, the light adapted, cone-driven ERG responses did not differ between *R91W;Nrl*^*-/-*^*;Elovl4*^*mut*^ and *R91W;Nrl*^*-/-*^ mice and declined in both strains similarly in an age-dependent manner (Fig 5A and 5B). This age-dependent loss of PR cells and decline in function is most likely caused by the intrinsic degeneration in the retina of all-cone mice, as described earlier [39].

## Discussion

Several mouse models have been generated to investigate the link between disease causing mutations in the *Elovl4* gene and macular degeneration that characterizes STGD3 pathophysiology [33–35,37]. None of these models showed the early onset cone PR degeneration observed in STGD3 patients but rather had rod PR cells affected. To examine the role of mutant *Elovl4* in cone PR degeneration [5,6], we intercrossed the *Elovl4* 5-bp deletion knock-in mouse as a model for STGD3 with the *R91W;Nrl*^*-/-*^strain which has an all-cone retina. The generated cone-only *R91W;Nrl*^*-/-*^*;Elovl4*^*mut*^ mouse carrying the human mutation should mimic the human disease affecting the macular cones in STGD3 patients, allowing to study the effect of mutant ELOVL4 protein specifically on cone function and survival.

Although the human *ELOVL4* mutation leads to cone PR cell death and macular atrophy in patients [5,6], the *R91W;Nrl*^*-/-*^*;Elovl4*^*mut*^ mouse did not show the anticipated early onset retinal degeneration. Both retinal function and morphology, analyzed up to one year of age, showed no differences between the control and *R91W;Nrl*^*-/-*^*;Elovl4*^*mut*^ mice, neither at early time points nor in aged mice. This preservation of retinal morphology and function despite the presence of mutant ELOVL4 was surprising, since rod-dominant transgenic mouse models indeed showed signs of retinal degeneration [33–35]. Furthermore, the original mutant mouse *Elovl4*^*tm1Rayy*^ exhibited preferential loss of S-cones [33] which is the predominant type of cones in the *Nrl*^*-/-*^ [40] and *R91W;Nrl*^*-/-*^ [39] mice. Therefore, the aggravated degeneration was expected in the S-cone-rich retinas of *R91W;Nrl*^*-/-*^*;Elovl4*^*mut*^ mice.

However, even though cones remained unaffected, the *Elovl4* mutation clearly reduced levels of mRNA transcript and ELOVL4 protein in *R91W;Nrl*^*-/-*^*;Elovl4*^*mut*^ mice. Most importantly, synthesis of VLC-PUFAs was severely diminished. Analysis of the PC-VLC-PUFA composition in the retina revealed reduction of the longest VLC-PUFAs already at early time points. E.g. in 6-week-old *R91W;Nrl*^*-/-*^*;Elovl4*^*mut*^ mice VLC-PUFAs—36:4, 36:5 and 36:6—were down to 19%, 29% and 42% of the controls, respectively. Yet, despite this early and substantial loss of retinal VLC-PUFAs, cones were not discernably affected. The PC-VLC-PUFA fraction makes 1.6% of total PC in 6-week-old *R91W;Nrl*^*-/-*^. This amount is reduced to 1.0 mol % PC in the age-matched *R91W;Nrl*^*-/-*^*;Elovl4*^*mut*^. When compared to data reported for the human macula (0.2 mol % PC [50]) or for rod-dominant mouse retinas (2.3 mol % PC [50]) this means that PC-VLC-PUFAs in the all-cone mice are reduced compared to rod-dominant mouse retinas, but are also still much higher than in the human macula. It remains to be determined if the plentiful reserves of VLC-PUFAs in *R91W;Nrl*^*-/-*^*;Elovl4*^*mut*^, and mice in general, might be responsible for the lack of phenotypic characteristics of STGD3 macular dystrophy.

On the other hand, haploinsufficency [9] or even an almost complete PR specific depletion of VLC-PUFAs does not induce PR degeneration [30,36]. Even though the two groups that analyzed the effects of *Elovl4* knock-down in the PR report somewhat conflicting results on cone function [30,36], they present data consistent with preservation of cone morphology upon near-total depletion of retinal VLC-PUFAs. This supports the concept that the mutant ELOVL4 protein, and not the reduced VLC-PUFAs, is the driving force for the observed PR degeneration in STGD3 patients and mouse models. It has been shown that mutant ELOVL4 mislocalizes to the outer segments of PR where it potentially induces PR cell death [51]. This goes along with the observed positive correlation between expression levels of mutant ELOVL4 and the severity of retinal degeneration in transgenic mouse models expressing increasing amounts of the human mutant ELOVL4 protein (TG1, TG1-2, TG2 and TG3) [34,35]. Yet, as VLC-PUFAs have been implicated in the maintenance of the structural and functional integrity of retinal PRs [11,31,32], it remains a puzzle why cones in mouse models do not follow the cone fate seen in STGD3 patients carrying the same mutation. It yet has to be determined which specific genetic, physiological and / or environmental factors could be responsible for the lack of human-like ELOVL4 effect on cone pathology in mice.

One trigger for cone degeneration in STGD might be lipofuscin-induced toxicity to the RPE, as lipofuscin accumulation and RPE-atrophic lesions were observed in patients [52,53]. Lipofuscin, a predominant fundus fluorophore, accumulates in the RPE during the normal ageing process. We have not observed signs of increased autofluorescence or changes in the RPE of the *R91W;Nrl*^*-/-*^*;Elovl4*^*mut*^ mice (not shown). The absence of lipofuscin accumulation in *R91W;Nrl*^*-/-*^*;Elovl4*^*mut*^ mice may not be surprising as the R91W mutation in RPE65 leads to hypomorphic enzymatic function of RPE65 i.e. reduced regeneration of the visual pigment [41]. A fully operational visual cycle, however, may be needed for the increased accumulation of lipofuscin in the RPE, as RPE65 patients show reduced fundus autofluorescence and *Rpe65*^*-/-*^ mice reduced lipofuscin content [54–56]. In that respect, it would be interesting to analyze the impact of *Elovl4* mutations in rod dominant R91W or *Rpe65*^*-/-*^ mutant mice. Of special interest would be one of the mutant ELOVL4-overexpressing lines (TG2 or TG3, see above) which show a strong degeneration [34]. Monitoring retinal morphology in e.g. *Rpe65*^*-/-*^;TG3 double mutant mice could help to answer the question if and to which extent lipofuscin accumulation might contribute to PR degeneration in mice and STGD3 patients.

Another conceivable reason for cones being spared in mice and not in humans may be the possibility that rod PRs in the parafoveal region of patients might be the first affected cells and that foveal cones are perturbed only in turn as a consequence of defective rods in STGD3. Although it still needs to be determined if defects in parafoveal rods could be a triggering event for STGD3 maculopathy, work by Curcio and colleagues [57] demonstrated that this may be the case for AMD. In AMD the pathological sequence of events starts with an initial degeneration of parafoveal rods leading to loss of support for the neighboring foveal cones and their degeneration, ultimately leading to deterioration of fine acuity vision. Such a scenario, though possible in STG3 patients, cannot be easily tested in rod-dominant mouse models lacking a macula, or for that matter, in all-cone mouse models lacking rods such as the *R91W;Nrl*^*-/-*^ mouse. The improvement of noninvasive imaging techniques, such as adaptive optics scanning laser ophthalmoscopy may help to discern rod from cone structure and may allow to determine the state or rods and cones in patients in the near future. This will be instrumental for a better understanding of the processes leading STGD3 disease.

Taken together, we report that the 5-bp deletion in *Elovl4* reduces retinal VLC-PUFA content but does not affect cone PR morphology or function in *R91W;Nrl*^*-/-*^*;Elovl4*^*mut*^ mice. As haploinsufficency or near total depletion of VLC-PUFAs in rods or cones—as discussed above [9,30,36]—has no impact on PR survival, this implies that i) VLC-PUFAs are dispensable for PR function and maintenance and that ii) deleterious effects are mediated by the truncated ELOVL4 protein directly. However, despite the presence of the mutation, the *R91W;Nrl*^*-/-*^*;Elovl4*^*mut*^ mice showed no sign of accelerated cone degeneration. Experiments on transgenic mice expressing different amounts of the truncated *ELOVL4* transgene imply that the degree of degeneration might be directly proportional to expression levels of the mutant protein [16,35]. Since both mice and STGD3 patients are heterozygous for *ELOVL4*, we hypothesize that the human macula has either a higher turnover rate of ELOVL4 protein than the mouse retina and/or is physiologically more sensitive to the presence of the mutant protein. High metabolic demand, excessive oxygen consumption and exposure to light make the human macula especially vulnerable to oxidative stress [58]. All of these factors may potentially increase the burden on macular cones and, in combination with the truncated misguided ELOVL4 protein, may be the crucial contributors to STGD3 pathology.

## Supporting information

S1 Fig Supplementary gene expression data analyzed by semiquantitative real-time PCR. (**A)** mRNA levels of G protein subunit alpha transducin 2 (*Gnat2*), arrestin 3 (*Arr3*), POU class 4 homeobox 1 (*Pou4f1*, alias: *Brn3a*), visual system homeobox 2 (*Vsx2*, alias: *Chx10*) expressed relative to 6-week-old *R91W;Nrl*^*-/-*^ mice. (**B)** mRNA levels of total (left) and *wt* (right) *Elovl4* in indicated strains expressed relative to 12-week-old *R91W;Nrl*^*-/-*^ mice. Expression was normalized to *Actb*. Shown are means ± SD. n = 3.
(TIF)

S1 Table Detailed results of retinal lipid and FA composition analysis. Phosphatidylcholine composition (sheet: S1a_Table_PC), phosphatidylethanolamine composition (sheet: S1b_Table_PE), phosphatidylserine composition (sheet: S1c_Table_PS), total lipid fatty acid composition (sheet: S1d_Table_TL-FA) in retinas of *R91W;Nrl*^*-/-*^*;Elovl4* and *R91W;Nrl*^*-/-*^ mice. n = 4.
(XLSX)

S2 Table NC3Rs ARRIVE guideline checklist. (PDF)
