# Supplementary figures and images for: Elovl4 5-bp deletion does not accelerate cone photoreceptor degeneration in an all-cone mouse
Source: PLoS One. 2018 Jan 2;13(1):e0190514. doi: 10.1371/journal.pone.0190514 (PMC5749830; doi:10.1371/journal.pone.0190514)

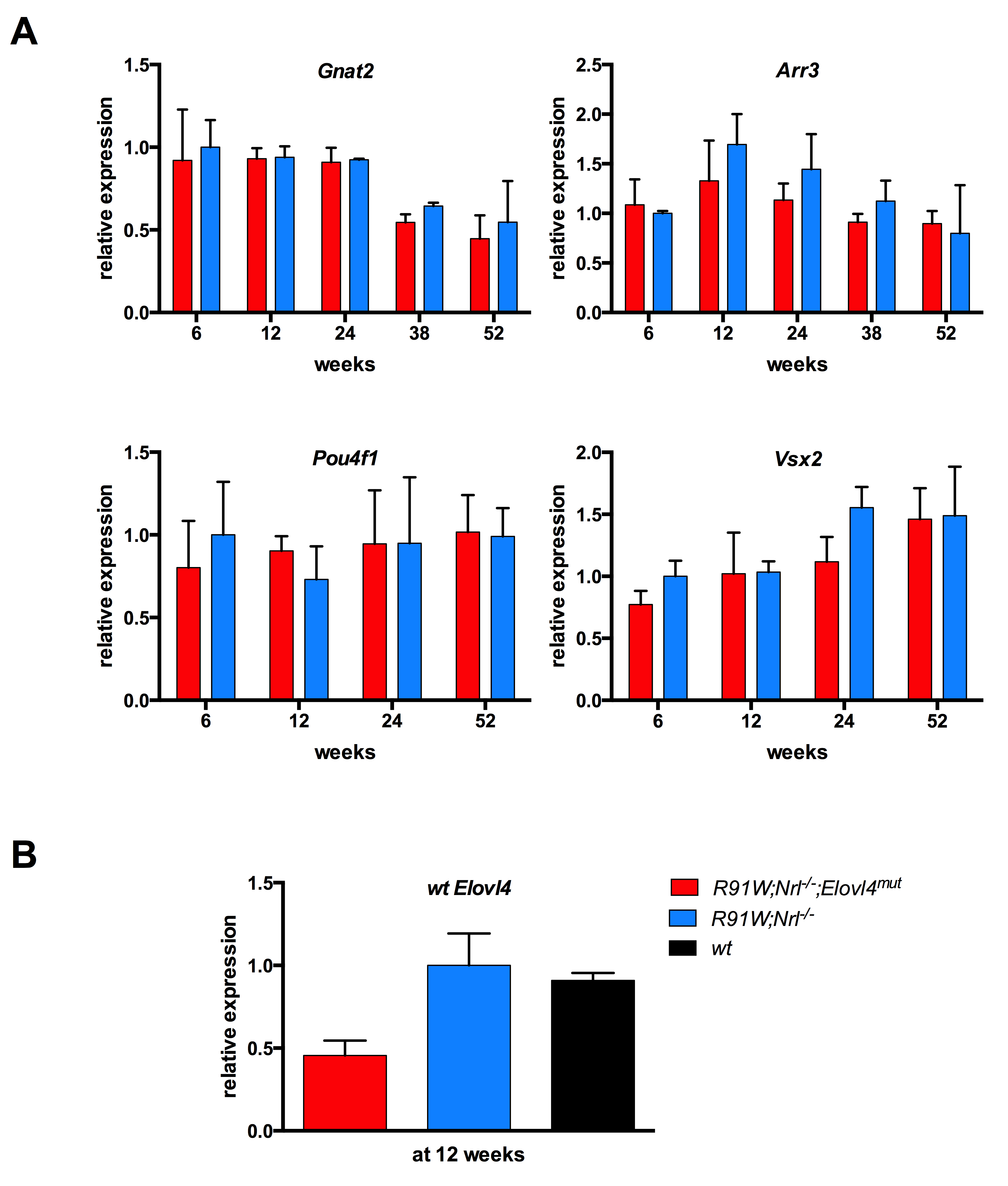

Supplement: S1 Fig — (A) mRNA levels of G protein subunit alpha transducin 2 (Gnat2), arrestin 3 (Arr3), POU class 4 homeobox 1 (Pou4f1, alias: Brn3a), visual system homeobox 2 (Vsx2, alias: Chx10) expressed relative to 6-week-old R91W;Nrl-/- mice. (B) mRNA levels of total (left) and wt (right) Elovl4 in indicated strains expressed relative to 12-week-old R91W;Nrl-/- mice. Expression was normalized to Actb. Shown are means ± SD. n = 3. (TIF) [file pone.0190514.s001.tif]
